# The complete mitogenome of nereid worm, *Neanthes glandicincta* (Annelida: Nereididae)

**DOI:** 10.1080/23802359.2017.1361346

**Published:** 2017-07-31

**Authors:** Geng-Ming Lin, Gilbert Audira, Chung-Der Hsiao

**Affiliations:** a Laboratory of Marine Biology and Ecology, Third Institute of Oceanography, State Oceanic Administration, Xiamen, China;; b Department of Bioscience Technology, Chung Yuan Christian University, Chung-Li, Taiwan;; c Center for Biomedical Technology, Chung Yuan Christian University, Chung-Li, Taiwan;; d Center for Nanotechnology, Chung Yuan Christian University, Chung-Li, Taiwan

**Keywords:** Nereid worm, mitogenome, phylogeny

## Abstract

In this study, the complete mitogenome sequence of nereid worm, *Neanthes glandicincta* (Annelida: Nereididae), has been decoded for the first time by PCR amplification and Sanger sequencing methods. The overall base composition of *N. glandicincta* mitogenome is 31.5% for A, 22.2% for C, 14.5% for G and 31.7% for T, and has GC content of 36.7%. The assembled mitogenome, consisting of 16,126 bp, has unique 13 protein-coding genes (PCGs), 22 transfer RNAs and 2 ribosomal RNAs genes. The complete mitogenome of *N. glandicincta* shows 65% identities to *Namalycastis abiuma*. All PCGs, tRNA and rRNA genes were encoded on H-strand. The potential D-loop is 1625 bp in length and located between tRNA-Gly and tRNA-Met. The complete mitogenome provides essential and important DNA molecular data for further phylogenetic and evolutionary analysis for Annelida.

Polychaetes are bristle-bearing segmented worms that belong to phylum *Annelida* and class Polychaeta. They are being the most dominant groups in benthic infaunal communities and they contribute about 80% to the total macrobenthic community and their diet includes microbial (fungi, protists, microalgae and bacteria), meiobial and organic substance. Polychaetes form a key component in the marine food chain mainly for bottom fish and some mammals as they establish an important source of food for demersal fish. Polychaetes are also being used towards biomonitoring programs worldwide as organic pollution indicators to survey the health status of the coastal marine environment. *Neanthes* are one of the most crucial groups of polychaete in coastal sediments, which lead an important role on the nutrient cycling in coastal sediments. Many species of this genus show a particular preference for littoral and supralittoral zones in association with decaying vegetation including mangroves, the strand area on beaches and inland waters such as sinkholes and riverbanks (subterranean waters) (Musale and Desai 2011; Li 2016).

*Neanthes glandicincta* is large species of polychaetes living in the intertidal mudflat of some bays in Asia. It is an economically important species for food source of birds, fishes and fishing bait and also an indicator species of environmental pollution. Moreover, this species shows a high tolerance to the contaminants with the wide distribution among the whole mudflat. This organism was mainly responsible for the temporal and spatial variations in infaunal communities in the intertidal mudflat. Previous study has showed that *N. glandicincta* inhibited abundant microbes in the gut, and these intestinal microbes indicate obvious axial distribution in diverse gut sections. The difference in microbial community structure in the gut sections describes the physicochemical conditions in the distinctive habits (Cai et al. 2013; Li 2016). In the present study, we used PCR amplification and Sanger sequencing methods to decode the complete mitogenome of *N. glandicincta* for the first time.

Samples (voucher no. 497) of *N. glandicincta* were collected from the Mangrove wetland of Yuandang Lake in Xiamen, China. We used PCR amplification and Sanger sequencing technology to decide the complete mitogenome of *N. glandicincta*. Primers designed to match generally conserved regions of target mtDNA were used to amplify short fragments from *16s*, *12s*, *cox1*, *atp6, cox3, nad4, cytB* and *nad1*. Specific primers were designed based on these conserved regions sequences and used to amplify the remained mtDNA sequence in several PCRs (Figure S1). PCR products were cloned into pMD18-T vector (Takara, Tokyo, Japan) and then sequenced by ABI 3730 automatic sequencer. Sequences were assembled by software of Geneious R9 and adjusted manually to generate the complete sequence of mitogenome.

The complete mitochondrial genome of *N. glandicincta* was 16,126 bp in size (GenBank KY094478) and its overall base composition is 31.5% for A, 22.2% for C, 14.5% for G and 31.7% for T, and has GC content of 36.7%, showing 65% identities to the complete mitogenome of *Namalycastis abiuma* (GenBank KU351089). The protein-coding, rRNA and tRNA genes of *N. glandicincta* mitogenome were predicted by using DOGMA (Wyman et al. 2004), ARWEN (Laslett and Canback 2008), MITOS (Bernt et al. 2013) tools and manually inspected. The complete mitogenome of *N. glandicincta* includes unique 13 protein-coding genes (PCGs), 22 transfer RNA genes and 2 ribosomal RNA genes (Figure 1). All PCGs, tRNA and rRNA genes were encoded on H-strand. It is important to note that 5 PCGs started with ATG codon (ATP8, COX1, COX2, ND3 and ND4), 3 with ATA codon (ATP6, ND4L and ND6), 2 with ATT codon (CYTB and ND5), 2 with ATC codon (ND1 and ND2) and 1 with GTG codon (COX3). Nine of 13 PCGs are inferred to terminate with TAA (ATP6, ATP8, CYTB, COX1, COX2, ND2, ND4, ND4L and ND5) and other 4 PCGs with TAG (COX3, ND1, ND3 and ND6) stop codon. Among 13 PCGs, the longest one is ND5 gene (1683 bp), whereas the shortest is ATP8 gene (192 bp). The size of small ribosomal RNA (12S rRNA) and large ribosomal RNA (16S rRNA) genes is 826 bp and 1203 bp, respectively. The potential D-loop is 1625 bp in length and located between tRNA-Gly and tRNA-Met.

**Figure 1.**
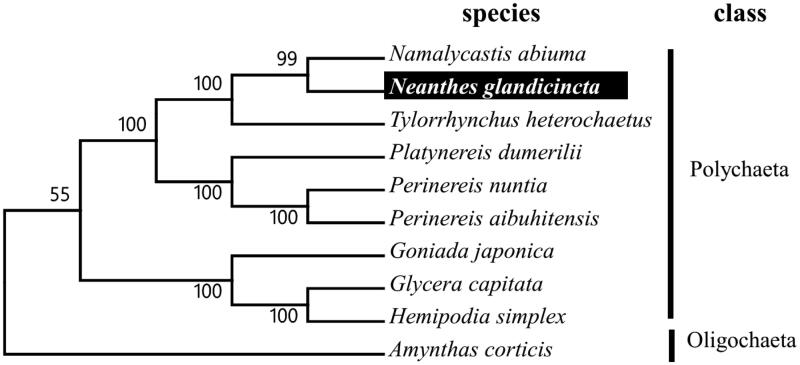
Molecular phylogeny of *N. glandicincta* and related species in Polychaeta based on complete mitogenome. The complete mitogenomes are downloaded from GenBank and the phylogenic tree is constructed by maximum likelihood method with 500 bootstrap replicates. The gene's accession number for tree construction is listed as follows: *Amynthas corticis* (NC_027832), *Hemipodia simplex* (KT989322), *Glycera capitata* (KT989319), *Goniada japonica* (KP867019), *Perinereis aibuhitensis* (KF611806), *Perinereis nuntia* (JX644015), *Platynereis dumerilii* (NC_000931), *Tylorrhynchus heterochaetus* (KM111507), *N. glandicincta* (KY094478) and *Namalycastis abiuma* (KU351089).

To validate the phylogenetic position of *N. glandicincta,* we used MEGA6 software (Tamura et al. 2013) to construct a maximum likelihood tree (with 500 bootstrap replicates) containing complete mitogenomes of 10 species derived from Polychaeta. *Amynthas corticis* (Black Wriggler) derived from Oligochaeta was used as out-group for tree rooting. Result shows *N. glandicincta* can be unambiguously grouped in Polychaeta showing close relation to *Namalycastis abiuma* with high bootstrap value supported (Figure 1). In conclusion, the complete mitogenome of the *N. glandicincta* deduced in this study provides essential and important DNA molecular data for further phylogenetic and evolutionary analysis for nereid worm phylogeny.

## Supplementary Material

TMDN_A_1361346_Supplementary_Information.zip
