# Supplementary material for: The complete mitogenome of nereid worm, Neanthes glandicincta (Annelida: Nereididae)
Source: Mitochondrial DNA B Resour. 2017 Jul 31;2(2):471–2. doi: 10.1080/23802359.2017.1361346 (PMC7799960; doi:10.1080/23802359.2017.1361346)
Supplement: TMDN_A_1361346_Supplementary_Information.zip [file TMDN_A_1361346_SM2282.zip › TMDN_A_1361346_Supplementary_Information.docx]

SCF1: CGGTCCCCACAGGCATCAAAGT

SCR1: CTGGCCGTTGGATGATAAACCC

SCF2: AACCGCACAGTTCTTCCAGT

SCR2: TATCAGCGATGGAGAAGGGG

SCF3: ACATGAGCTCATCACAGCCT

SCR3: GCGAATATGGCTACTACACCTC

SCF4: TTATGCCAAATGTACTTGGAG

SCR4: CTGCGCTTATGTTAGCCGCCAG

SCF5: TTATCAGCAATAGCCTACGACC

SCR5: GGTAGTTGGGTTAAACTGTGGC

SCF6: TGCCATTCCTCTATCCCT

SCR6: GAAGTTTTACTAGGAACGCCAT

SCF7: CCATCACCGCAAGTCTTCCC

SCR7: GACGGGCGATGTGTGCACATC

SCF8: AAAGTGCAGCTAACGGTTGA

SCR8: GTCTCTGATCATCCCAATC

SCF9: TTCTGTCTCAACTGTGCGC

SCR9: GTTTGAGCGATGCTTCGTAG

SCF10: TACCCTCACTCAGCCTCCCC

SCR10: GGGCTGATGAGGAGGGATC

SCF11: GGAAGCTTGCAATAGCCATG

SCR11: AAAGATGGCTAGGTCTACGGAG

SCCOX1F: CCAGACATAGCCTTTCCACG

SCCOX1R: GTAGCCCACTTCCACTATGT
